# Effectiveness of Juvenile *Eriocheir sinensis* in Controlling *Pomacea canaliculata* and Their Growth and Nutritional Response to Feeding on the Snail

**DOI:** 10.3390/ani15010085

**Published:** 2025-01-02

**Authors:** Jie Wang, Yixiang Zhang, He Lv, Weiqi Shen, Weiping Fang, Rongfei Zhang, Hanqu Zhao, Qiang Sheng

**Affiliations:** 1 Zhejiang Province Key Laboratory of Aquatic Resources Conservation and Development, College of Life Sciences, Huzhou University, Huzhou 313000, China; wangjiecg2022@163.com (J.W.); yxzhang@zjhu.edu.cn (Y.Z.); 02592@zjhu.edu.cn (H.L.); rongfeizhang@zjhu.edu.cn (R.Z.); 02469@zjhu.edu.cn (H.Z.); 2 Changxing County Aquatic and Agricultural Machinery Center, Huzhou 313100, China; 13732377757@163.com (W.S.); fangweiping2004@126.com (W.F.)

**Keywords:** juvenile *Eriocheir sinensis*, *Pomacea canaliculata*, biological control, amino acid and fatty acid

## Abstract

In recent years, *Pomacea canaliculata* has aggressively invaded rice fields in Asia, resulting in significant agricultural losses. Biological control can effectively reduce the damage caused by *P. canaliculata*. This research evaluates *E. sinensis* as a biocontrol for *P. canaliculata*, focusing on its feeding preferences and optimal control density on snails of three sizes, as well as the effects on the nutritional quality of juvenile crabs post consumption. Our findings reveal that juvenile *E. sinensis* exhibit a strong preference for feeding on small snails, effectively managing populations at densities of 600 snails per tank. Crab feeding significantly reduces the survival and activity of snails. Furthermore, consumption of *P. canaliculata* meat alters the crabs’ physiology. Female crabs show elevated levels of molting hormones, total energy yield (TEY), and condition factor (CF), while males demonstrate increased lipid, moisture, and TEY levels. The amino acid profiles shift, with higher isoleucine and leucine levels in female hepatopancreas and reduced histidine in the muscles. Notably, the total Σn-3 PUFA content in female muscles fed on snail meat exceeds that of those given commodity grain. This study underscores the dual benefits of employing juvenile *E. sinensis* for managing *P. canaliculata* while enhancing crab farming practices.

## Simple Summary

The rice–crab co-culture system has generated substantial economic benefits for agriculture in Asia. However, recent years have seen the invasion of *Pomacea canaliculata*, which has caused significant damage to rice seedlings. This underscores the importance of determining whether *E. sinensis* can effectively control this invasive species. This study analyzes the following: (1) the biological control effects of juvenile *E. sinensiss* on *P. canaliculata*; and (2) the impact of consuming *P. canaliculata* on the growth and nutritional value of juvenile crabs. This study found that juvenile *E. sinensis* can consume small, medium, and large *P. canaliculata*, and their feeding efficiency is highest at maximum densities. Following consumption, changes occur in the nutritional composition within their bodies. This research is significant for the management of *P. canaliculata* in rice fields and for promoting the application of rice–crab co-culture systems.

## 1. Introduction

*Pomacea canaliculata*, commonly known as the apple snail, belongs to the class Gastropoda, phylum Mollusca, and family Ampullariidae, within the genus *Pomacea* [1]. Native to the Amazon River basin in South America, this species has spread globally [2], including in North America, Europe, East Asia, Southeast Asia, and the Pacific Islands, due to its strong adaptability, rapid reproduction, high consumption rate, and lack of natural predators [3]. It has been listed as one of the 100 most harmful invasive alien species by the International Union for Conservation of Nature (IUCN) [4]. In addition, its excretory and secretory products can pollute water quality and contribute to eutrophication in aquatic ecosystems [5].

In China, *P. canaliculata* is widely distributed in the southern and southeastern regions of the Yangtze River and is expanding northward due to climate change [6]. Surveys have shown that the damage rate in rice fields ranges from 7% to 15%, with some areas reporting losses as high as 64%, particularly in Guangxi, Guangdong, Hainan, and Zhejiang [7]. To mitigate these losses, current control strategies include physical, chemical, and biological methods. Biological control, which uses parasitic or predatory organisms to maintain pest populations at manageable levels, is considered effective, environmentally friendly, and economically viable [8]. Aquatic species such as the eider (*Tadorna tadorna*) [9], carp (*Cyprinus carpio*) [10], Chinese soft-shelled turtle (*Pelodiscus sinensis*) [11], and wide-bodied golden-line leech (*Whitmania pigra*) [12] have all been explored for this purpose. The size variations in *P. canaliculata* influence the foraging choices of these predators [13]. *T. tadorna* primarily preys on small- and medium-sized snails, while *P. sinensis* feeds on snails of all sizes, including small, medium, and large.

*Eriocheir sinensis*, a key economic species in China [14], offers potential as a biological control agent due to its wide diet, which includes small shrimp, fish, worms, insects, shellfish, and aquatic plants [15]. In many respects, crabs can act as biological control agents for snails. Cespedes et al., in 2024, observed that the blue crab uses its powerful claws to crack open the shells of adult *P. canaliculata* and consume them [16]. The northern mud crab can track and prey on *Tritia obsoleta* by sensing the signals left behind by the species [17]. Previous studies have shown that *E. sinensis* can effectively consume *B. quadrata* [18] and *B. aeruginosa* [19], a practice which promoted their growth and improved feed efficiency in those studies [20]. When *E. sinensis* are fed a mixed diet of snails and artificial feed, they exhibit better weight gain, improved hypoxia tolerance, and reduced feed costs [21,22].

The adoption of rice–crab symbiotic systems, where crabs are raised in paddy fields, has grown in recent years. These systems represent a sustainable agricultural practice by providing natural habitats and food for crabs while crab feces fertilize rice plants, enriching soil nutrients and enhancing rice yield [23]. This approach aligns with the concept of Integrated Multi-Trophic Aquaculture (IMTA), a modern form of polyculture which optimizes resource use and promotes ecological balance in aquaculture systems [24]. Additionally, *E. sinensis* contributes to pest control, reducing reliance on chemical pesticides [25]. Given the overlap in food resources and habitat between *E. sinensis* and *P. canaliculata*, a potential predation relationship may exist. If *E. sinensis* can effectively consume *P. canaliculata* of various sizes, it could serve as a biological control agent, mitigating the population growth of this invasive species and reducing its environmental impact. Furthermore, as *E. sinensis* exhibits its fastest growth during the juvenile stage, investigating this phase is critical to understanding its development and assessing its potential role in controlling *P. canaliculata*.

During the growth of juvenile crabs, gender influences both growth performance and the changes in internal nutrient composition. Female crabs exhibit a higher digestive efficiency than males, requiring the accumulation of substantial nutrients during rapid development [26]. Furthermore, females reach maturity earlier than males, and throughout the juvenile growth period, they show elevated hormone levels. Therefore, investigating the impact of gender on juvenile crabs’ consumption of *P. canaliculata* and their growth is essential.

There is a hypothesis that juvenile *E. sinensis* can consume *P. canaliculata* of various sizes, effectively controlling the population growth of the snails and reducing environmental harm. Based on this hypothesis, this study aims to evaluate the biological control potential of juvenile *E. sinensis* on *P. canaliculata* by addressing the following specific objectives: 1. feeding behavior and impact, to investigate the feeding preferences of juvenile *E. sinensis* for different sizes of *P. canaliculata*, assess how crab predation influences the snails’ own feeding activity, and evaluate the associated changes in water quality parameters under different treatment conditions; 2. biological control efficiency, to determine the efficiency of *E. sinensis* in reducing *P. canaliculata* populations under varying densities and establish its potential role in pest management within rice–crab symbiotic systems; and 3. growth and nutritional effects, to analyze the impact of consuming *P. canaliculata* on the growth performance, amino acid profiles, and fatty acid composition of juvenile crabs. The findings will provide valuable insights into the dual benefits of *E. sinensis* as an effective biological control tool and its capacity to enhance the sustainability and economic value of rice–crab farming systems.

## 2. Materials and Methods

### 2.1. Experimental Organisms

Different sizes of *P. canaliculata* were collected from farmland in Qianjin Town, Huzhou City, Zhejiang Province, and reared in aquaculture water from the breeding center. The snails were classified into three size categories based on shell height: small snails, 0 mm ≤ H < 5 mm; medium snails, 5 mm ≤ H < 30 mm; and large snails, 30 mm ≤ H < 60 mm. The collected snails were reared in four 70 L plastic tanks (46 cm × 30 cm × 28 cm) at the Aquatic Biological Breeding Experimental Center of Huzhou Normal University, with each tank equipped with an aeration stone. Seven days prior to the experiment, the water temperature was stabilized at 25.0 ± 0.5 °C, with a 12 h light/12 h dark photoperiod. Each tank was provided with approximately 20 g of lettuce daily at 8:00 AM. Waste and uneaten food were removed using a small suction device, and dead snails were promptly removed. Additionally, one-third of the tank water was replaced every day. Feeding was discontinued three days before the experiment began.

Juvenile *E. sinensis* crabs with a shell height of 22 ± 3.0 mm were purchased from Shuixuan Modern Agriculture Co., Ltd., Huzhou, China. Male and female crabs were reared separately in six 300 L plastic tanks (100 cm × 65 cm × 60 cm) with a water depth of 6 cm for one week. During this period, the water temperature was maintained at 25.0 ± 0.5 °C, with a 12 h light/12 h dark cycle. The study was conducted in two stages, requiring two separate purchases of crab juveniles. The first experiment represented the first stage, while the second and third experiments constituted the second stage. In the first experiment, 40 medium-sized snails were introduced daily into each tank for three days before the experiment to allow the crabs to adapt to the predation environment. Before the second and third experiments, the crabs underwent a three-day starvation period to stimulate predatory behavior. Throughout the holding period, daily suction cleaning and water changes were performed to maintain water quality in all tanks.

### 2.2. Experimental Design

#### 2.2.1. The Biological Control Efficacy of Juvenile *E. sinensis* on *P. canaliculata*

##### Experient 1: Biological Control Effects of Juvenile *E. sinensis* on Different Sizes of *P. canaliculata*

**Figure 1:**
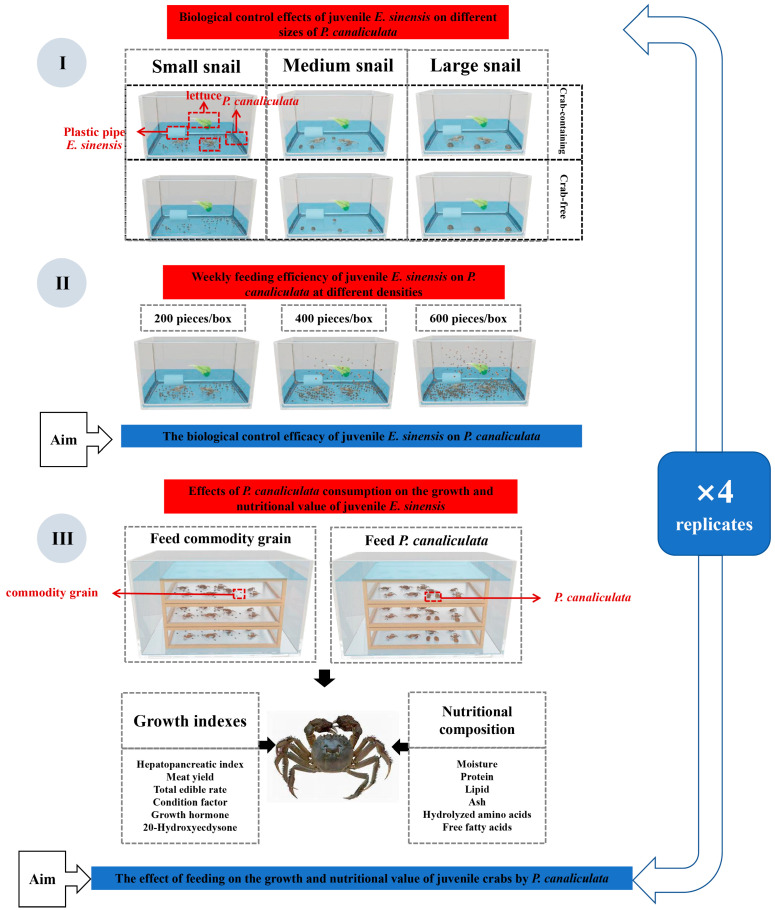
Experimental design flowchart. The formal experiment used the same conditions as the temporary rearing period. (**I**) **Experiment 1:** The experiment included two treatments: crab presence, with levels “crab-containing” and “crab-free”, and snail size, categorized into three levels—small, medium, and large. Each tank contained the following: 5 g lettuce, a plastic tube, one male and one female crab, and 100, 5, or 3 snails (small, medium, and large). Each combination of crab presence/non-presence and snail size was replicated four times. (**II**) **Experiment 2:** The experiment had one treatment, density, with three levels: 200, 400, and 600. Each tank housed one male and one female crab, with snail densities of 200 (low), 400 (medium), or 600 (high). Each level of density was replicated four times. (**III**) **Experiment 3:** Eight tanks were split into two feeding treatments—the feeding commodity grain group and the feeding *P. canaliculata* meat group. Each tank had 3 baskets, holding 4 male and 4 female crabs, fed either 5 g of commodity grain or *P. canaliculata* meat.

This experiment was conducted from 11 March to 10 April 2024, in a 2 × 3 factorial design using 24 identical 70 L plastic tanks. The first factor was the presence of crabs, with two levels: “crab-containing” and “crab-free”. The second factor was the size of the *P. canaliculata* snails offered as feed, with three levels: small (100 snails), medium (5 snails), and large (3 snails). Each combination of crab presence and snail size was replicated four times. In the “crab-containing” treatment, 12 tanks were stocked with one male and one female juvenile *E. sinensis* to minimize aggression and mortality among the crabs. The remaining 12 tanks were designated as the “crab-free” treatment, serving as the controls. This factorial arrangement allowed for the evaluation of not only the individual effects of each factor—crab presence and snail size—but also the interaction between these two factors (Figure 1(I)).

The water depth in each tank was maintained at approximately 6 cm. Daily waste was removed, to prevent food shortages due to the rapid feeding rates of crabs in certain tanks, which could affect the experimental results, and, to maintain consistency across the experiment, 0.2 g of commodity grain (equivalent to 3% of the crabs’ body weight) was provided per feeding. The number of surviving, damaged, and dead snails in each tank was recorded. Based on the feeding rate of juvenile crabs, the initial snail density was restored every 7 days. Additionally, 5 g of lettuce was provided every three days based on snail consumption, with any uneaten lettuce removed and replaced with fresh lettuce. The leftover lettuce was dried in an 80 °C oven to a constant weight for analysis, and the snails were weighed at the initial and final time (every 3 days). To minimize crab mortality, 1/3 of the water was replaced every five days, based on preliminary results. Water quality parameters, including temperature, pressure, salinity, redox potential, conductivity, pH, and dissolved oxygen, were continuously monitored throughout the experiment using a Pro Plus handheld multiparameter water quality analyzer. Additionally, TN, TP, and NH_3_-N concentrations were analyzed using the HACH Flow Injection Analyzer QC8500 (Hach Company, Loveland, CO, USA). The formal experiment used the same conditions as the temporary rearing period (Figure 1(I)).

##### Experient 2: Weekly Feeding Efficiency of Juvenile *E. sinensis* on *P. canaliculata* at Different Densities

Building on the insights from Experiment 1, which demonstrated a preference for small snails, Experiment 2 focused solely on this size category. The study employed a single-factor design to examine the effects of snail density on the feeding behavior of *E. sinensis*. In accordance with the principle of maximum application [27], snail density was tested at three levels—low (200 snails per tank), medium (400 snails per tank), and high (600 snails per tank). Conducted over 30 days, from 11 April to 11 May 2024, the experiment utilized 12 identical 70 L plastic tanks, with each density level replicated four times. Daily snail consumption was recorded, and snail populations were replenished weekly to maintain the initial density levels. The experimental setup replicated the conditions of the preliminary rearing period to ensure consistency and reliability. This design enabled the evaluation of the effects of varying snail densities on feeding rates, offering insights into optimal conditions for biological control (Figure 1(II)).

#### 2.2.2. The Effect of Feeding on *P. canaliculata* on the Growth and Nutritional Value of Juvenile Crabs Experient 3: Effects of *P. canaliculata* Consumption on the Growth and Nutritional Value of Juvenile *E. sinensis*

This experiment aimed to assess the effects of *P. canaliculata* consumption on the growth and nutritional value of juvenile *E. sinensis*, conducted, as shown in Figure 1(III), over a 30-day breeding cycle in eight 300 L plastic tanks (100 cm × 65 cm × 60 cm). The study used a 2 × 2 factorial design considering two factors: the type of feed and the gender of the crabs. The feed types were two—commodity grain and *P. canaliculata* meat—and the sex levels were male and female. Four tanks were designated to receive commodity grain as feed, and four tanks were fed *P. canaliculata* meat. The nutritional composition of the commodity grain included the following: crude protein 36%, crude fiber 8%, crude fat 5%, crude ash 18%, total phosphorus 1%, moisture 12%, and lysine 1.6%. Each feed level was replicated four times, resulting in a total of eight tanks, with each tank containing three wooden baskets (25 cm × 20 cm × 10 cm), holding a total of twenty-four juvenile *E. sinensis* crabs (eight crabs per basket). Each basket had an equal number of four males and four females to evaluate the potential differential effects of feed on the growth and nutritional uptake by gender. The baskets were interconnected, allowing water to flow between them and ensuring a uniform water quality throughout the tank. Each tank was fed 5 g of *P. canaliculata* meat or 5 g of commodity grain every three days. Larger live *P. canaliculata* were crushed, their shells removed, and fresh snail meat was fed immediately. Water levels were maintained to cover the baskets, and one-third of the water was replaced every three days to ensure optimal environmental conditions. The baskets were perforated to facilitate water exchange and prevent crabs from escaping, while still allowing natural interactions among the crabs within each basket. Any remaining *P. canaliculata* meat was weighed and recorded to adjust the feed input as needed. The formal experiment used the same conditions as the temporary rearing period.

At the end of the breeding period, all crabs were weighed. From each basket, one male and one female crab were selected for body composition analysis, while the remaining crabs were anesthetized on ice, bled, and stored in 2 mL centrifuge tubes at −20 °C. Additionally, hepatopancreas and muscle tissues were collected, weighed, and preserved at −80 °C for a subsequent nutritional analysis (Figure 1(III)).

### 2.3. Indicator Analysis

#### 2.3.1. Growth Indicators of *P. canaliculata*

The growth indicators for *P. canaliculata* included survival rate, damage rate, feeding rate, and maximum feeding rate. The formulas for their calculation were as follows [28,29]:

Survival rate = [(N_1_ − N_0_)/N_0_] × 100% (1)

Damage rate = N_2_/N_0_ × 100% (2)

Feeding rate = N_3_/N_0_ × 100% (3)

(4) $$\mathrm{Maximum}\;\mathrm{feeding}\;\mathrm{rate}=\frac{2F}{t(M0+M1)}$$

In the above, N_0_ represents the initial number of *P. canaliculata* at the start of the experiment, N_1_ denotes the number of surviving *P. canaliculata*, N_2_ indicates the number of damaged *P. canaliculata*, and N_3_ is the number of *P. canaliculata* consumed. F stands for the total amount of lettuce consumed by *P. canaliculata* during the experiment, *t* is the duration of the experiment, and M_0_ and M_1_ are the total weight of *P. canaliculata* before and after the experiment, respectively.

#### 2.3.2. Growth Performance and Nutritional Quality Indicators of Juvenile *E. sinensis*

At the end of Experiment 3, the final weights of all juvenile *E. sinensis* were measured using an electronic scale. The following performance and nutritional quality indicators were calculated [30]:

Weight gain rate (WGR) = [(W_t_ − W_0_)/W_0_] × 100%, (5)

Hepatopancreatic index (HIS) = W_H_/W_t_ × 100%, (6)

Meat yield (MY) = W_M_/W_t_ × 100% (7)

Condition factor (CF) = W_t_/CL^3^ (8)

Total edible yield (TEY) = MY+ HSI (9)

In the formulas, t represents the duration of the experiment, and W_0_ and W_t_ denote the total weight of *E. sinensis* before and after the experiment, respectively. W_H_ is the wet weight of the hepatopancreas of *E. sinensis*, W_M_ refers to the weight of the dissected muscle, and CL refers to the carapace length of juvenile *E. sinensis* crabs, measured in centimeters (cm).

The growth hormones and 20-hydroxyecdysone levels in the blood were measured using an ELISA kit from the Jiangsu Province [31]. Whole crabs were used to assess moisture, ash, lipid, and protein content. The moisture content was determined by drying samples at 105 °C for 48 h until a constant weight was reached. The protein content was measured using an Elementar Dumas nitrogen analyzer (Elementar Analysensysteme GmbH, Langenselbold, Germany), while the lipid content was assessed using the Soxhlet extraction method. The ash content was determined through high-temperature ashing [32,33]. Amino acid and fatty acid analyses were performed on the hepatopancreas and muscle tissues of both male and female *E. sinensis*. Amino acids were analyzed using a Hitachi L8900 amino acid analyzer [34], and the fatty acid profiles were determined by gas chromatography–mass spectrometry (GC–MS) (Agilent Technologies, Santa Clara, CA, USA) [35].

### 2.4. Data Statistics

All measurement data were rounded to three decimal places and analyzed using SPSS 25.0 and R 4.3.3. Due to the non-parametric nature of the data in Section 2.2.1, the biological control effects of juvenile *E. sinensis* on *P. canaliculata* of three size categories—specifically survival rate, maximum feeding rate, breakage rate, and juvenile crab feeding rate—were analyzed using the Aligned Rank Transform (ART) Mixed Model. The Friedman repeated means test was used to evaluate the effects of time on these parameters. Similarly, the effects of invasion density and time on feeding quantity were analyzed using the Friedman repeated means test.

Water quality data were first analyzed using Pearson correlation to identify variables with low correlations. Non-correlated variables were then tested for differences between crab-containing and crab-free treatments using Kruskal–Wallis tests. Significant variables were visualized using box plots to demonstrate their distribution between treatments. This approach ensures that only the most independent and statistically significant factors are used for interpretation, avoiding potential redundancy in highly correlated variables.

Comparisons of growth performance (including weight gain, hormones, hepatosomatic index (HIS), muscle yield (MY), total energy yield (TEY), and condition factor (CF)) and nutritional quality indicators (including protein, lipid, ash content, and moisture) between the *P. canaliculata* meat group and the commodity feed group were conducted using the ART method. Comparisons of hydrolyzed amino acids and free fatty acids between groups were performed using the Mann–Whitney U test. The ART Mixed Model was also applied to assess the effects of gender on amino acids and fatty acids. Graphs were generated using R 4.3.3 and GraphPad Prism 8.

## 3. Results

### 3.1. Biological Control Effects of Juvenile E. sinensis on Different Sizes of P. canaliculata

**Figure 2:**
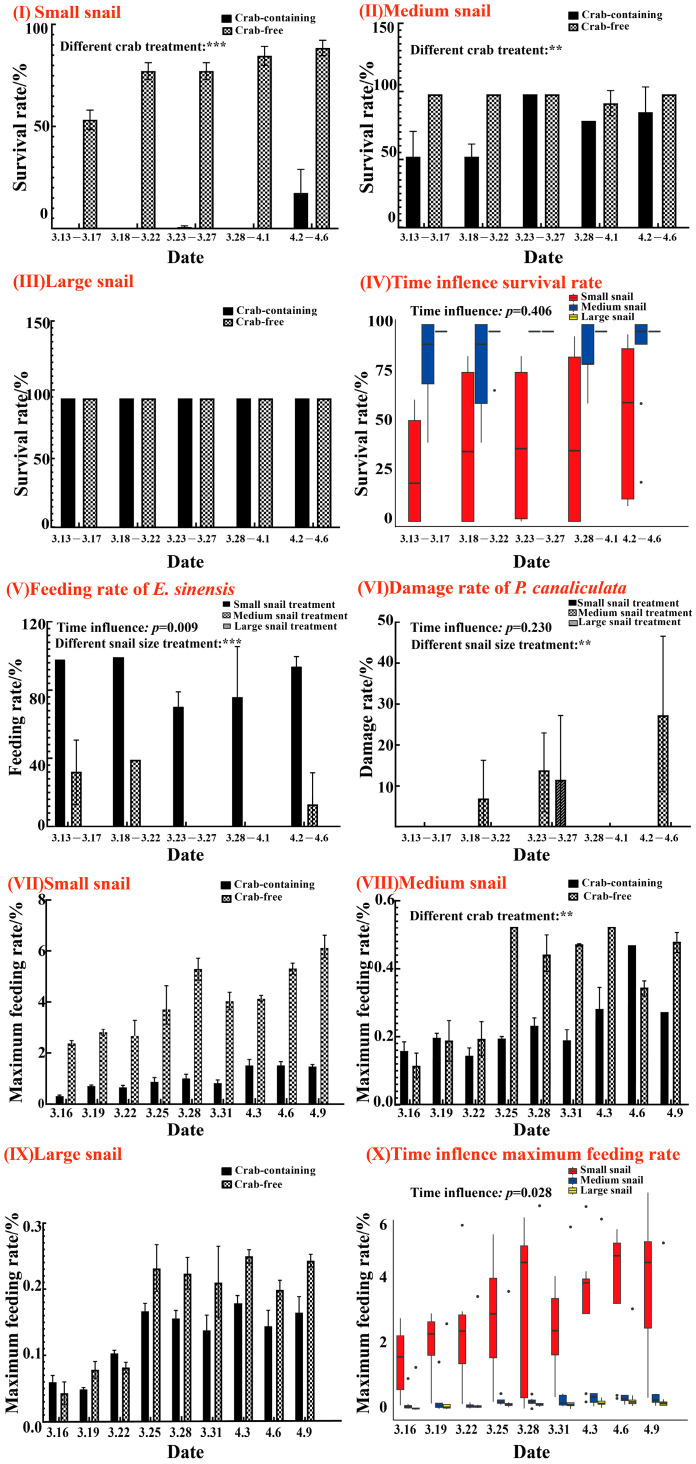
Comparison of the effect of two crab treatments on the survival rate and maximum feeding rate of *P. canaliculata*, and the effects of snail size on the feeding rate of juvenile crabs and the damage rate of snails under the crab treatments. Graphs are represented in the form of (Mean ± SD) deviation. The horizontal line in the box plot indicates that the data lack standard deviation and can only be represented as a short straight line, while the dots on the box plot represent individual observations. (**I**)–(**III**) Effects of crab treatments on the survival rates of three sizes of snails; (**IV**) Impact of the time on the survival rate of *P. canaliculate*; (**V**,**VI**) The influence of snail size on the feeding rate of juvenile crabs and the changes in damage rates of three snail sizes under feeding impac; (**VII**)–(**IX**) Effects of crab treatments on the maximum feeding rates of three sizes of snails; (**X**) Impact of the time on the maximum feeding rate of *P. canaliculata*. *p* < 0.0001 ***, *p* < 0.001 **.

Juvenile *E. sinensis* significantly reduced the survival rates of small- and medium-sized *P. canaliculata.* As shown in Figure 2(I,III), the survival rates in the “crab-containing” treatment were significantly lower than in the “crab-free” treatment for both the small-sized snail group (F = 138.18, *p* < 0.0001) and the medium-sized snail group (F = 9.964, *p* = 0.003). The size of the snails had a significant impact on survival (F = 99.774, *p* < 0.0001), with survival rates ranked as large snail > medium snail > small snail, indicating that smaller snails had lower survival rates. Through the Friedman repeated measures test, it was found that the effect of time on the survival rate of *P. canaliculata* was weak (*p* = 0.406), indicating that time cannot be considered a key factor influencing the survival rate. Instead, the primary consideration should be the impact of snail size treatment on the survival rate.

The size and specifications of *P. canaliculata* also significantly influenced the feeding rates (F = 99.774, *p* < 0.0001) (Figure 2(IX)). In the “crab-containing” treatment, small snails were consumed at a higher rate than medium and large snails every five days. Except for the large-sized snail group, the feeding pattern followed a fluctuating trend of increase–decrease–increase, with the lowest feeding rates recorded between 23 March and 2 April. The small-sized snail group had a consumption rate of 71.670 ± 9.030%, while the medium- and large-sized snail groups showed no consumption during this period (Figure 2(IX)). With time as a categorical variable, the Friedman repeated measures test revealed that time had a significant effect on the feeding rate of *E. sinensis* (*p* = 0.009). The damage rate also varied significantly based on snail size (F = 5.342, *p* = 0.007). The medium-sized snail group exhibited the highest damage rate, peaking at 26.670 ± 18.860% between 2 April and 7 April, while the small-sized snail group showed a damage rate of 0.000%. However, the Friedman repeated measures test revealed that time had little effect on the damage rate (*p* = 0.230).

Juvenile *E. sinensis* also reduced the feeding rates of *P. canaliculata* on lettuce. Across all size groups, the maximum feeding rates were lower in the “crab-containing” treatment than in the “crab-free” treatment, with significant differences observed in the medium-sized snail group (F = 5.106, *p* = 0.027). The maximum feeding rates across different size treatments were as follows: small snail > medium snail > large snail (crab-containing: F = 21.223, *p* < 0.0001; crab-free: F = 68.075, *p* < 0.0001). The Friedman repeated measures test revealed that time had a significant effect on the maximum feeding rate (*p* = 0.028).

**Figure 3:**
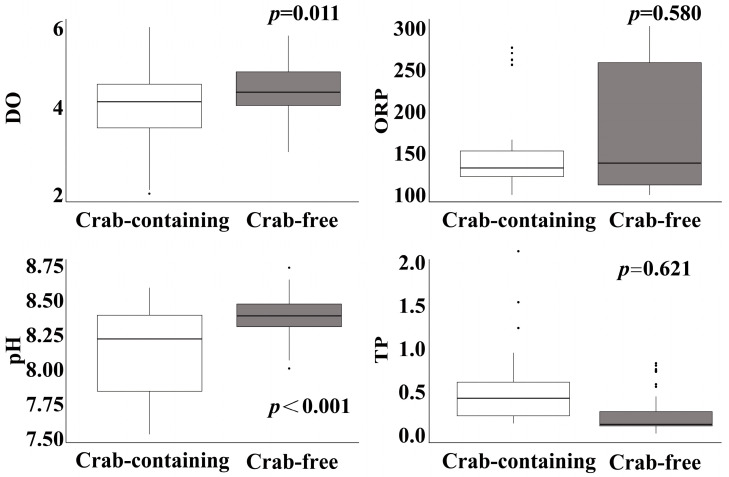
Box plots for uncorrelated variables tested with Kruskal–Wallis. The monitored parameters include the following: dissolved oxygen (DO); oxidation-reduction potential (ORP); pondus hydrogenii (pH); and total phosphorus (TP). Graphs are represented in the form of (Mean ± SD) deviation, while the dots on the box plot represent individual observations.

**Figure A1:**
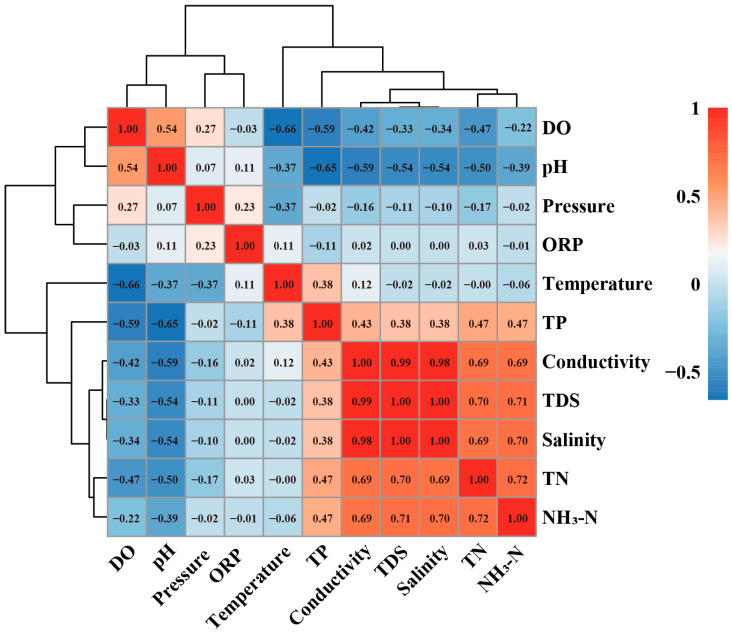
Correlation heatmap of water quality indicators. The monitored parameters include the following: temperature; pressure; dissolved oxygen (DO); conductivity; total dissolved solids (TDSs); salinity; pondus hydrogenii (pH); oxidation-reduction potential (ORP); total nitrogen (TN); total phosphorus (TP); and ammonia nitrogen (NH_3_-N).

Water quality variables were analyzed for correlations (Figure A1), and only variables with correlation coefficients |r| ≤ 0.7 were tested using Kruskal–Wallis tests to assess their differences between crab-containing and crab-free treatments. Dissolved oxygen (DO) (*p* = 0.011) and pH (*p* < 0.0001) were found to be significantly higher in the crab-free treatment (Figure 3). In contrast, ORP (*p* = 0.580) and TP (*p* = 0.621) showed no significant differences between the treatments (Figure 3).

### 3.2. Weekly Feeding Efficiency of Juvenile E. sinensis on P. canaliculata at Different Densities

**Figure 4:**
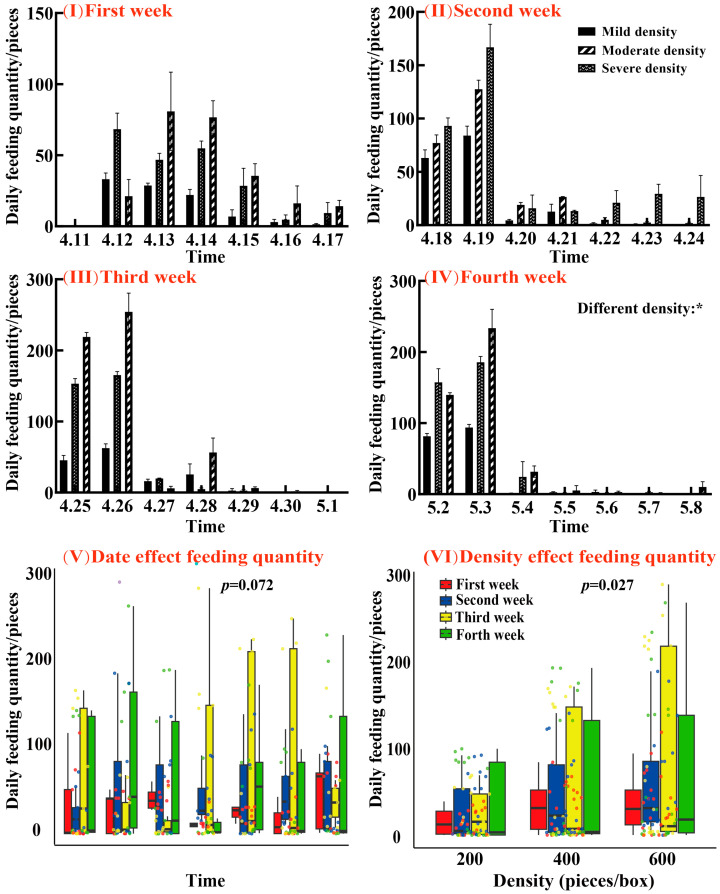
A: Feeding efficiency of juvenile *E. sinensis* under three levels of snail invasion from 1 November to 1 May, broken down by weekly intervals. Graphs are represented in the form of (Mean ± SD) deviation, where the dots on the box plot represent individual observations: (**I**) first week; (**II**) second week; (**III**) third week; and (**IV**) fourth week. The linear model shows (**V**) the effects of different dates on the crab’s feeding rate, while (**VI**) shows the effect of different densities of *P. canaliculata* on the crab feeding rate. *p* < 0.01 *.

When the density was set at 600 snails per tank, juvenile *E. sinensis* exhibited the highest daily feeding efficiency in the fourth week, significantly surpassing the performance of groups with 400 and 200 snails per tank, as highlighted in Figure 4(IV). This observation indicates a close relationship between an increased prey density and an enhanced feeding activity, with significant differences (F = 3.615, *p* = 0.032). The analysis using the Friedman repeated measures test, as shown in Figure 4(V), revealed that temporal changes during the study had a minimal impact on the feeding rates (*p* = 0.072). In contrast, changes in snail density had a more pronounced effect on the feeding rates (*p* = 0.027). This finding was supported by Figure 4(VI), further confirming that prey density, rather than temporal factors, is the critical determinant of feeding behavior.

### 3.3. Impact of Feeding on P. canaliculata on the Growth Performance and Nutritional Composition of Juvenile E. sinensis

**Figure 5:**
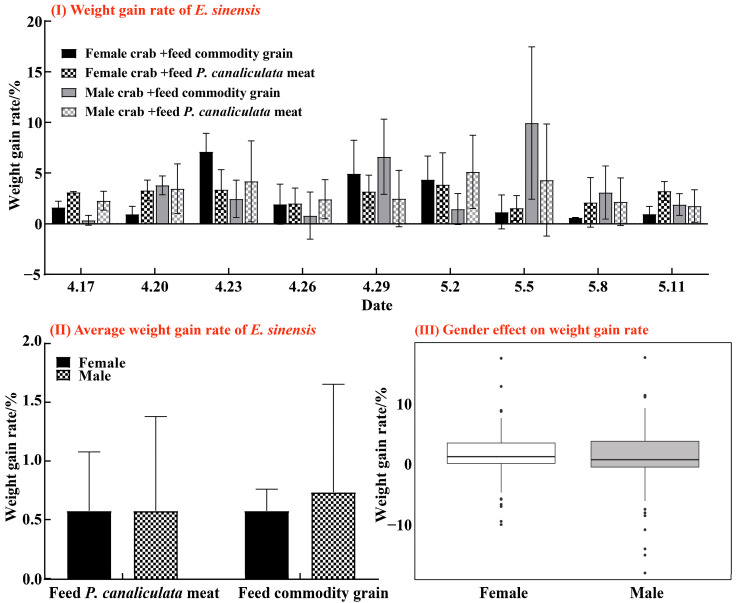
Changes in the body weight growth rate of female and male juvenile *E. sinensis* every 3 days under two feeding methods. Graphs are represented in the form of (Mean ± SD) deviation, where the dots on the box plot represent individual observations: (**I**) body weight growth rate of female and male juvenile *E. sinensis* under two feeding methods; and (**II**) average weight gain of male and female juvenile crabs under the two feeding methods. Meanwhile, the linear model shows (**III**) the effect of gender on the weight gain rate.

The weight gain rate of juvenile *E. sinensis* fed with *P. canaliculata* meat was slightly higher than that of the commodity grain group, though the difference was not statistically significant (*p* = 0.488). For female crabs, the highest weight gain rate in the *P. canaliculata* meat group was 7.114 ± 1.801% on 23 April, while the commodity grain group peaked at 3.855 ± 3.148% on May 2nd. In male crabs, the maximum growth rate for the *P. canaliculata* meat group reached 9.941 ± 7.519% on 5 May, compared to 5.113 ± 3.618% for the commodity grain group on 2 May (Figure 5(I)). In summary, the results showed that the weight gain rate for female crabs in the commodity grain group was the same as that in the *P. canaliculata* meat group, whereas male crabs exhibited a 1.6% higher weight gain rate in the *P. canaliculata* meat group compared to the commodity grain group (Figure 5(II)). Additionally, the effect of gender on the weight growth rate was relatively small (F = 0.235, *p* = 0.630) (Figure 5(III)).

The meat yield (MY) and the total edible yield (TEY) of male *E. sinensis* in the *P. canaliculata* meat group were significantly higher than those in the commodity grain group (MY: F = 0.745, *p* = 0.004; TEY: F = 4.618, *p* = 0.048,). Similarly, for female crabs, the TEY and condition factor (CF) in the *P. canaliculata* meat group were significantly higher than in the commodity grain group (CF: F = 27.532, *p* = 0.030; TEY: F = 41.928, *p* = 0.004). No significant differences were observed for other growth performance indicators. Gender had a significant effect on the TEY (F = 2.793, *p* < 0.001).

In terms of proximate composition, male crabs in the commodity grain group exhibited higher moisture and lipid contents than those in the *P. canaliculata* meat group (moisture: F = 44.619, *p* = 0.050; lipid: F = 21.384, *p* = 0.050), while their ash content was lower (F = 49.368, *p* = 0.050). However, no significant differences in proximate composition were found among the female crabs across the two feeding treatments. Gender had a less significant effect on the ash, moisture, protein, and lipid content in juvenile *E. sinensis* (F = 0.482, *p* = 0.488).

**Figure 6:**
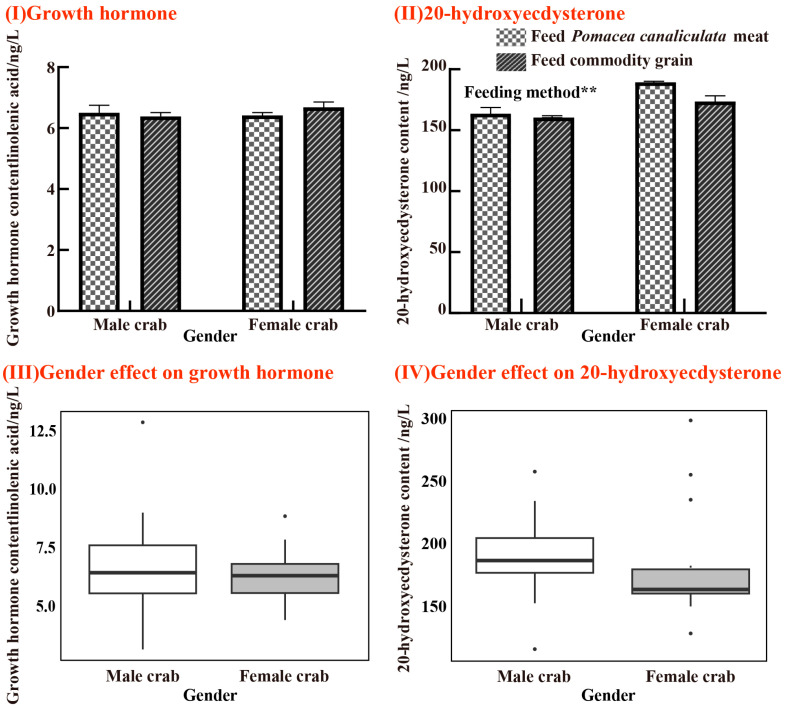
Variations in growth hormone and 20-hydroxyecdysterone levels in female and male juvenile *E. Sinensis* across two feeding regimens. Graphs are represented in the form of (Mean ± SD) deviation, where the dots in the box plot represent individual observations. *p* < 0.010 **. (**I**) Growth hormone and (**II**) 20-hydroxyecdysone. Meanwhile, the linear model shows the (**III**) effect of gender on the growth hormone and (**IV**) the effect of gender on 20-hydroxyecdysone.

Regarding growth hormone levels, female crabs in the commodity grain group showed slightly higher levels than those in the *P. canaliculata* meat group. In contrast, male crabs fed with *P. canaliculata* meat exhibited higher growth hormone levels than those in the commodity grain group. However, these differences in growth hormone levels were not statistically significant (*p* > 0.050) (Figure 6(I)). The changes in 20-hydroxyecdysterone (20E) and growth hormone levels suggest that female crabs in the *P. canaliculata* meat group had higher 20E levels compared to the commodity grain group (F = 43.618, *p* = 0.050) (Figure 6(II)), while no significant differences were observed in male crabs. Gender significantly influences the levels of 20-hydroxyecdysone (F = 5.731, *p* = 0.022) (Figure 6(IV)).

In the female juvenile *E. sinensis* group, the levels of isoleucine (*p* = 0.050), leucine (*p* = 0.050), and histidine (*p* = 0.050) and the EAA/TAA ratio (essential amino acids to total amino acids) (*p* = 0.050) in the hepatopancreas were higher in the *P. canaliculata* meat group compared to the commodity grain group. However, the histidine levels in the muscle were lower in the *P. canaliculata* meat group (*p* = 0.050). No significant differences were observed in other amino acids (*p* > 0.050). Regarding the total amino acid content, in female crabs’ hepatopancreas, *P. canaliculata* meat group > commodity grain group. Regarding muscle, *P. canaliculata* meat group < commodity grain group. In male crabs’ hepatopancreas, *P. canaliculata* meat group < commodity grain group; meanwhile, regarding muscle, *P. canaliculata* meat group > commodity grain group (Table A1). Gender had a significant effect on the amino acid content in the hepatopancreas and muscle of juvenile crabs (hepatopancreas: F = 8.762, *p* = 0.047; muscle: F = 7.682, *p* = 0.049).

The total fatty acid analysis showed that, in female juvenile *E. sinensis*, the hepatopancreas of the *P. canaliculata* meat group had higher levels of polyunsaturated fatty acids (PUFAs) than the commodity grain group (*p* = 0.050). However, the Σn-3 PUFA and Σn-6 PUFA levels were lower in the *P. canaliculata* meat group (*p* = 0.050) (Appendix A Table A2).

In male crabs, the hepatopancreas of the *P. canaliculata* meat group exhibited a higher saturated fatty acid (SFA) content compared to the commodity grain group (*p* = 0.050), with polyunsaturated fatty acids being lower (*p* = 0.050). Notably, stearic acid (C18:0) was significantly higher in the *P. canaliculata* meat group (*p* = 0.050). Additionally, the DHA content within the Σn-3 PUFA category was significantly lower in the *P. canaliculata* meat group compared to the commodity grain group (Table A2).

In the muscle tissue, female crabs fed with *P. canaliculata* meat showed higher Σn-3 PUFA levels than those in the commodity grain group (*p* = 0.050). In contrast, male crabs in the commodity grain group had higher levels of monounsaturated fatty acids (MUFAs) and DHA compared to those in the *P. canaliculata* meat group (*p* = 0.050). However, the n-3/n-6 ratio and highly polyunsaturated fatty acid levels were lower in the *P. canaliculata* meat group (*p* = 0.050) (Table A2). However, gender had less of an effect on the fatty acid content (F = 5.273, *p* = 0.617).

## 4. Discussion

### 4.1. The Biological Control Effect of Juvenile E. sinensis on P. canaliculata

In this study, juvenile *E. sinensis* were found to consume *P. canaliculata*, providing a new approach for controlling snail populations using crustaceans. Hooks et al. [36] found that *D. sayi*, a native predatory crab abundant on the east coast of the United States, is capable of consuming *L. saxatilis*. Peura et al. found that juvenile hermit crabs can consume *C. fornicata*. Hermit crabs pry intact juvenile snails from the substrate and then consume the juveniles, shell and all [37]. Shaked et al. found that both male and female crayfish are effective predators of snails, with females showing slightly better predation rates [38].

Research suggests that the size preferences of *E. sinensis* when preying on *P. canaliculata* reflect an ecological adaptation strategy—selecting smaller snails can enhance the foraging efficiency, as they offer a higher energy return per unit of time while minimizing energy expenditure [39]. Larger snails, on the other hand, require more energy to consume and may potentially cause damage to the crab. In contrast, smaller snails provide a more accessible and efficient foraging option for the predator [40]. In this study, juvenile *E. sinensis* showed a clear preference for small snails over medium and large ones. Within five days, the feeding rate on small snails reached 100% (Figure 2), as the crabs could consume them whole without damaging them. In contrast, large snails were less frequently attacked due to their size, while the medium-sized snail group exhibited a higher damage rate compared to both the small-sized snail and large-sized snail groups (Figure 2). Time affected the feeding rate of juvenile crabs. Over time, snails exhibit defensive behaviors, such as retreating into their shells, influencing crab predation [41]. As a result, in this study, the feeding rate of juvenile crabs gradually decreased over time (Figure 2). This is consistent with the findings of Joe C. Gunn et al. [42], where different sizes of ramshorn snails were found to affect the feeding of Belostoma. Ramshorn snails exhibit behaviors of burrowing into the mud to evade predation, which increases their survival rate over time. Prawns are voracious predators of *P. canaliculata* of various sizes, especially smaller snails (up to 15 mm shell size), and the survival rate of the snails changes over time [43]. Small black carp primarily feed on small Angulyagra polyzonata, and predation significantly decreases as the snail size increases. Under the predation pressure from small black carp, *A. polyzonata* adjusts its shell hardness and exhibits escape behaviors; thus, the survival of *A. polyzonata* over time is affected [44].

Additionally, in aquatic ecosystems, predator–prey interactions not only influence the population and survival of prey but also affect their behavior and habitat selection [45]. The presence of crabs significantly reduced the feeding activity of *P. canaliculata*, particularly in the medium-sized snail group, demonstrating that juvenile *E. sinensis* can effectively control medium-sized snails. Sura et al. [46] demonstrated that the presence of crayfish reduces the feeding rate of native snails compared to situations without crayfish. Over time, the maximum feeding rate of *P. canaliculata* gradually increased across the different groups, suggesting that the snails adapted to the predation environment, leading to improved survival rates and, as a result, higher feeding rates. This is consistent with the research of Goeppner et al. [47], where, as time progressed, the feeding rate of freshwater snails gradually increased under the influence of bluegill sunfish, demonstrating characteristics of adaptation to the predatory environment.

During the predation process, interactions between predators and prey can lead to increased water turbidity, nutrient release, and reduced dissolved oxygen levels [48]. Water quality indicators are often interrelated and influence each other [49]. In this study, the presence of crabs led to lower dissolved oxygen (DO) and pH levels in the water. This suggests that crab predation not only disrupts the snails but also disturbs the water environment. These findings align with Guo et al. [50] (Figure 3), who observed that increased biomass from crab excretion leads to higher DO consumption and greater water acidity. Tang et al. [51] found that, in the water of ponds used for cultivating *E. sinensis*, the NH_3_-N concentration increased, while the DO and pH levels decreased. Similarly, Bi et al. [52] found that higher oxygen consumption by hybrid sturgeons increased CO_2_ production, thereby lowering the water pH. Dissolved oxygen not only affects pH but is also closely related to other indicators in the water [53]. In this study, dissolved oxygen shows a strong correlation with pressure, conductivity, total dissolved solids (TDSs), total nitrogen (TN), and ammonia nitrogen (NH_3_-N) changes. Additionally, total dissolved solids impact conductivity, which is consistent with Davidraj et al. [54].

As prey populations decline, predation efficiency decreases due to the reduced frequency of predator–prey encounters [55]. This study found that the optimal feeding efficiency occurred at a density of 600 snails per tank, indicating that higher densities of small snails within a manageable range enhanced the crabs’ daily feeding rates. These findings align with Stephan et al. [56], who demonstrated that prey density directly influences predation rates (Figure 4). Jacobsen et al. [57] discovered that piscivorous Eurasian perch Perca fluviatilis more easily finds food in environments with a higher number of prey, thereby increasing the frequency of predation events. Ortiz et al. [58] demonstrated that increasing prey density enhances the functional response (FR) and predation by paralarvae. The above studies all demonstrate that a higher prey density increases the predation rate of predators.

### 4.2. Impact of Feeding P. canaliculata on the Growth, Nutrition, and Development of Juvenile E. sinensis

The growth hormone plays a crucial role in accelerating protein synthesis in crabs, promoting cell division, growth, and a more efficient nutrient utilization, ultimately enhancing their overall development [59]. During growth, molting hormones fluctuate as energy accumulates, with molting being triggered when hormone levels peak [60]. The levels of growth hormone (GH) and 20-hydroxyecdysone (20E) in male and female *E. sinensis* differ significantly due to growth and reproductive demands [61]. In this study, female crabs fed *P. canaliculata* meat showed significantly higher levels of 20-hydroxyecdysone (20E) compared to the commodity grain group (Figure 6), indicating that the nutrient composition of *P. canaliculata* meat may better support molting processes in females. For male crabs, the growth hormone levels were slightly higher in the *P. canaliculata* meat group than in the commodity grain group, suggesting improved support for growth-related functions. Conversely, female crabs in the *P. canaliculata* meat group exhibited lower growth hormone levels compared to the commodity grain group, which may reflect the allocation of energy towards molting and reproductive processes rather than somatic growth. These findings highlight the differential hormonal responses of male and female crabs to dietary variations, emphasizing the role of diet in meeting gender-specific physiological demands.

Male and female *E. sinensis* exhibit significant differences in growth performance [62], which also affect the measurement of nutritional components under the two feeding methods. In terms of body weight growth, both male and female crabs in the *P. canaliculata* meat and commodity grain groups exhibited continuous weight gain. However, the meat yield (MY) and condition factor (CF) of female crabs, as well as the MY and total edible yield (TEY) of male crabs, were significantly higher in the *P. canaliculata* meat group (Table 1). These results indicate that feeding *P. canaliculata* meat can effectively promote the growth of female juvenile crabs.

Lipids are essential energy sources and enhance the flavor of crab meat [63]. In this study, male crabs fed with *P. canaliculata* meat exhibited a lower lipid and moisture content, but a higher ash content, compared to the commodity grain group. In contrast, female crabs showed no significant differences in nutritional composition, suggesting that commodity grain offers a more balanced nutrient profile. Thus, relying solely on *P. canaliculata* meat may not fully meet the developmental nutritional needs of juvenile crabs.

The hepatopancreas, gonads, and muscles are key edible tissues in *E. sinensis*, with their amino acid and fatty acid profiles serving as indicators of nutritional quality [64]. Female crabs fed with *P. canaliculata* meat had higher levels of isoleucine, leucine, histidine, and essential amino acids in the hepatopancreas, while muscle histidine levels were lower. In male crabs, threonine levels were higher in the *P. canaliculata* meat group (Table A1). Isoleucine and leucine are essential for liver cell repair and participate in protein synthesis [65]. Histidine supports liver and muscle regeneration and helps maintain acid–base balance [66], while threonine is involved in muscle tissue formation and immune enhancement [67]. These amino acids are primarily derived from meat, and their levels increase when crabs consume snail meat [68].

In this experiment, the concentration of saturated fatty acids in the hepatopancreas of juvenile crabs was found to be higher in the group fed on the meat of *P. canaliculata* compared to the commodity grain group (Table A2). The content of C18:0 is particularly significant among saturated fatty acids, as it is primarily involved in the synthesis of various bioactive substances and provides energy for the organism [69]. Consumption of *P. canaliculata* meat significantly elevates the levels of saturated fatty acids in the hepatopancreas; these fatty acids are absorbed and converted into body fat, subsequently being stored in the hepatopancreas [70]. This accumulation markedly influences the overall fatty acid profile in the hepatopancreas, resulting in increased concentrations of these fatty acids. Saturated fatty acids competitively inhibit metabolic pathways, thereby affecting the metabolism and utilization of unsaturated fatty acids, specifically Σn-3PUFA and Σn-6PUFA [71]. Consequently, the levels of Σn-3PUFA and Σn-6PUFA in the hepatopancreas of both male and female *E. sinensis* larvae were found to be lower than those in the commodity grain group.

### 4.3. Potential Application of E. sinensis for Controlling P. canaliculata Invasion

Rice–crab co-culture is primarily applied in China, leveraging the symbiotic relationship between rice and crabs to promote the flow of energy and matter, creating a virtuous cycle which benefits the growth of both rice and crabs [72]. River crabs prey on weeds, locusts, leeches, and other small aquatic pests in rice fields, reducing the incidence of field diseases [73]. This study demonstrated that *E. sinensis* can consume *P. canaliculata*, indicating that rice–crab co-culture can effectively control the invasion of these snails. Juvenile crabs’ preference for small snails can suppress the snails at their source, and investigations into invasion levels revealed that juvenile *E. sinensis* can significantly reduce the density of *P. canaliculata*. Research on the growth performance and nutritional quality of the crabs suggests that consuming *P. canaliculata* can significantly improve the quality of the crabs, showing promising application prospects. This approach would greatly reduce the damage caused by *P. canaliculata* to rice fields.

This study demonstrates the potential of juvenile *E. sinensis* as a biological control agent against *P. canaliculata* under laboratory conditions. Juvenile crabs exhibit strong feeding capabilities and adaptability, making them suitable for pest management in controlled environments. However, laboratory settings may not fully represent the complexities of rice field ecosystems. Practical application in rice–crab co-culture systems will require further field trials to evaluate their effectiveness under real-world conditions. Such trials would address variables such as prey density, water quality, and habitat structure, which are critical for assessing their long-term impact on *P. canaliculata* control.

Additionally, the current study focuses exclusively on juvenile crabs, leaving a gap in understanding how crabs at other developmental stages contribute to pest control. Adult crabs, with their stronger predation abilities, may offer complementary benefits. Investigating the interactions among crabs of different growth stages could provide a comprehensive view of their collective impact on *P. canaliculata* populations and inform best practices for aquaculture management.

Despite these promising results, the application of *E. sinensis* in rice–crab co-culture systems must be carefully managed to mitigate potential risks. There is a possibility of inadvertently spreading *P. canaliculata* through contaminated equipment or water systems during crab farming. Moreover, introducing crabs into new environments could pose zoonotic risks or disrupt local ecosystems if not properly regulated. To address these concerns, strict biosecurity measures and continuous monitoring must be implemented to ensure the sustainable and safe integration of *E. sinensis* in pest management programs.

By addressing these challenges and expanding research efforts, *E. sinensis* could become a valuable tool for controlling *P. canaliculata* in rice–crab co-culture systems, contributing to sustainable agriculture and enhanced aquaculture productivity.

## 5. Conclusions

Juvenile *E. sinensis* crabs exhibit a notable control effect on *P. canaliculata,* particularly concerning smaller snails, which are preferred as a food source. Their feeding behavior demonstrates the effective regulation of various densities of these small snails. This indicates that juvenile *E. sinensis* can serve as an effective biological control agent in aquatic ecosystems, particularly in rice–crab co-culture systems, where *P. canaliculata* poses a significant threat to rice production. By preying on *P. canaliculata,* juvenile crabs can reduce the population of this invasive pest, thereby minimizing crop damage and the need for chemical pesticides, contributing to a more sustainable and environmentally friendly farming system. In addition to controlling snail populations, juvenile crabs showed changes in molting hormones, amino acids, and fatty acids after consuming *P. canaliculata,* potentially enhancing energy reserves and disease resistance. These physiological changes further support their application in integrated pest management strategies, as healthier crabs with enhanced growth and resistance can provide economic benefits to farmers while maintaining ecological balance.

## Figures and Tables

**Table 1:** Growth performance and proximate composition of female and male juvenile *E. sinensis* under two feeding regimens.

	Female		Male	
Item	Commodity Grain	*P. canaliculata* Meat	Commodity Grain	*P. canaliculata* Meat
HIS (%)	5.891 ± 1.922	5.543 ± 1.924	5.976 ± 1.265	6.112 ± 2.132
MY (%)	3.778 ± 0.876	3.578 ± 1.545	**4.321 ± 1.448**	**4.945 ± 2.362**
TEY (%)	**6.758 ± 2.372**	**8.678 ± 2.330**	**7.978 ± 1.110**	**11.036 ± 4.131**
CF (g/cm^3^)	**49.314 ± 7.428**	**53.507 ± 8.418**	48.209 ± 9.213	54.935 ± 13.023
Moisture (%)	70.941 ± 0.319	70.774 ± 4.000	**75.907 ± 2.336**	**69.917 ± 2.336**
Protein (%)	33.488 ± 1.702	33.580 ± 4.000	36.044 ± 3.299	35.400 ± 2.336
Lipid (%)	27.546 ± 10.339	27.319 ± 3.189	**31.329 ± 13.334**	**30.889 ± 2.960**
Ash (%)	49.050 ± 1.535	51.483 ± 3.236	**47.618 ± 2.692**	**54.210 ± 3.234**

Bold black font indicates a significant difference.

## Data Availability

Data are contained within the article.

## Appendix A

**Table A1:** Comparison of key hydrolyzed amino acids in the hepatopancreas and muscles of female and male juvenile *E. sinensis* across two feeding regimens.

Hydrolyzed Amino Acids (mg/g)		Female		Male	
		Commodity Grain	*P. canaliculata* Meat	Commodity Grain	*P. canaliculata* Meat
Lle	HP M.	**0.340 ± 0.031** 0.697 ± 0.058	**0.794 ± 0.034** 0.639 ± 0.168	0.338 ± 0.079 0.533 ± 0.177	0.345 ± 0.07 0.665 ± 0.126
Leu	HP M.	**0.533 ± 0.056** 0.961 ± 0.081	**1.097 ± 0.405** 0.874 ± 0.215	0.517 ± 0.089 0.788 ± 0.186	0.507 ± 0.096 0.973 ± 0.161
Lys	HP M.	0.278 ± 0.033 0.758 ± 0.081	0.600 ± 0.297 0.690 ± 0.188	0.263 ± 0.056 0.586 ± 0.251	0.208 ± 0.031 0.820 ± 0.145
Met	HP M.	0.120 ± 0.017 0.282 ± 0.044	0.273 ± 0.126 0.203 ± 0.083	0.127 ± 0.045 0.230 ± 0.090	0.097 ± 0.043 0.271 ± 0.073
Phe	HP M.	0.284 ± 0.030 0.543 ± 0.043	0.559 ± 0.188 0.485 ± 0.130	0.268 ± 0.035 0.411 ± 0.115	0.262 ± 0.062 0.529 ± 0.093
Tyr	HP M.	0.245 ± 0.006 0.406 ± 0.040	0.345 ± 0.098 0.335 ± 0.110	0.230 ± 0.024 0.310 ± 0.121	0.256 ± 0.056 0.412 ± 0.081
Thr	HP M.	0.325 ± 0.026 0.463 ± 0.052	0.713 ± 0.312 0.382 ± 0.118	**0.272 ± 0.021** 0.381 ± 0.094	0.312 ± 0.076 0.448 ± 0.093
Val	HP M.	0.264 ± 0.050 0.513 ± 0.050	0.713 ± 0.330 0.455 ± 0.141	0.271 ± 0.039 0.405 ± 0.112	**0.241 ± 0.067** 0.500 ± 0.093
EAA	HP M.	2.390 ± 0.223 5.095 ± 2.038	5.095 ± 2.038 4.064 ± 1.145	2.287 ± 0.364 3.645 ± 1.138	2.287 ± 0.364 4.618 ± 0.856
Asp	HP M.	0.644 ± 0.102 1.108 ± 0.104	0.890 ± 0.467 0.924 ± 0.255	0.629 ± 0.155 0.867 ± 0.307	0.432 ± 0.037 1.096 ± 0.173
Ser	HP M.	0.254 ± 0.027 0.393 ± 0.027	0.402 ± 0.210 0.344 ± 0.095	0.200 ± 0.035 0.310 ± 0.102	0.166 ± 0.024 0.390 ± 0.081
Glu	HP M.	0.957 ± 0.112 1.870 ± 0.191	2.030 ± 0.834 1.772 ± 0.523	0.924 ± 0.181 1.558 ± 0.419	0.899 ± 0.165 1.939 ± 0.379
Gly	HP M.	0.493 ± 0.069 0.680 ± 0.04	0.994 ± 0.355 0.674 ± 0.125	0.422 ± 0.045 0.617 ± 0.103	0.446 ± 0.093 0.691 ± 0.105
Ala	HP M.	0.338 ± 0.015 0.853 ± 0.058	0.840 ± 0.430 0.826 ± 0.236	0.329 ± 0.107 0.681 ± 0.206	0.331 ± 0.037 0.848 ± 0.158
His	HP M.	0.172 ± 0.019 0.240 ± 0.009	0.368 ± 0.156 0.174 ± 0.038	0.135 ± 0.009 0.204 ± 0.037	0.157 ± 0.032 0.234 ± 0.048
Arg	HP M.	0.284 ± 0.084 0.673 ± 0.081	0.619 ± 0.282 0.511 ± 0.128	0.619 ± 0.282 0.492 ± 0.215	0.254 ± 0.082 0.693 ± 0.160
Pro	HP M.	0.040 ± 0.007 0.086 ± 0.016	0.087 ± 0.037 0.083 ± 0.024	0.041 ± 0.004 0.062 ± 0.021	0.035 ± 0.005 0.097 ± 0.034
NEAA	HP M.	3.182 ± 0.340 5.904 ± 0.512	6.231 ± 2.616 5.307 ± 1.401	2.953 ± 0.542 4.791 ± 1.405	2.720 ± 0.428 5.987 ± 1.093
TAA	HP M.	5.572 ± 0.546 10.529 ± 0.931	11.326 ± 4.653 9.371 ± 2.545	5.240 ± 0.900 8.45 ± 2.543	4.947 ± 0.900 10.606 ± 1.946
EAA/TAA	HP M.	0.429 ± 0.012 0.498 ± 0.235	0.452 ± 0.006 0.433 ± 0.006	0.438 ± 0.016 0.431 ± 0.005	0.495 ± 0.181 0.435 ± 0.004
EAA/NEAA	HP M.	0.753 ± 0.036 0.890 ± 0.420	0.825 ± 0.019 0.763 ± 0.018	0.780 ± 0.050 0.756 ± 0.015	0.887 ± 0.300 0.771 ± 0.014

Bold black font indicates a significant difference. HP—hepatopancreas; M—muscle; Lle—isoleucine; Leu—leucine; Lys—lysine; Met—methionine; Phe—phenylalanine; Tyr—tyrosine; Thr—threonine; Val—valine; EAAs—essential amino acids; Asp—aspartic acid; Ser—serine; Glu—glutamic acid; Gly—glycine; Ala—alanine; His—histidine; Arg—arginine; Pro—proline; NEAAs—non-essential amino acids; and TAAs—total amino acids.

**Table A2:** Comparison of major fatty acids in the hepatopancreas and muscles of female and male juvenile *E. sinensis* across two feeding regimens.

		Female		Male	
Fatty Acids (%)		Commodity Grain	*P. canaliculata* Meat	Commodity Grain	*P. canaliculata* Meat
C4:0	HP M.	**0.777 ± 0.032** 0.004 ± 0.001	**0.296 ± 0.003** 0.004 ± 0.00	**0.560 ± 0.170** 0.006 ± 0.00	**0.256 ± 0.048** 0.01 ± 0.00
C6:0	HP M.	1.434 ± 0.079 -----	1.343 ± 0.056 -----	**1.586 ± 0.215** -----	**1.207 ± 0.024** -----
C8:0	HP M.	**2.078 ± 0.085** -----	**1.920 ± 0.061** -----	1.970 ± 0.160 -----	1.853 ± 0.101 -----
C10:0	HP M.	**2.791 ± 0.241** -----	**0.573 ± 0.041** -----	**2.616 ± 0.081** -----	**1.749 ± 0.081** -----
C11:0	HP M.	**2.658 ± 0.093** -----	**0.628 ± 0.043** -----	1.518 ± 0.086 -----	1.621 ± 0.076 -----
C12:0	HP M.	**1.621 ± 0.118** -----	**0.449 ± 0.017** -----	**0.807 ± 0.080** -----	**0.978 ± 0.034** -----
C13:0	HP M.	1.074 ± 0.077 -----	1.081 ± 0.034 -----	**1.725 ± 0.142** -----	**1.454 ± 0.057** -----
C14:0	HP M.	3.955 ± 0.045 -----	4.068 ± 0.292 -----	**4.225 ± 0.047** -----	**4.050 ± 0.071** -----
C15:0	HP M.	**1.907 ± 0.059** -----	**1.559 ± 0.041** -----	**1.482 ± 0.008** -----	**1.368 ± 0.033** -----
C16:0	HP M.	**16.594 ± 0.472** **1.808 ± 0.130**	**14.154 ± 0.699** **0.824 ± 0.07**	14.767 ± 0.205 **0.955 ± 0.028**	14.465 ± 0.311 **0.731 ± 0.009**
C18:0	HP M.	**4.951 ± 0.045** **4.394 ± 0.175**	**18.192 ± 0.451** **4.849 ± 0.007**	**5.085 ± 0.622** **3.866 ± 0.01**	**17.851 ± 0.042** **4.849 ± 0.006**
C20:0	HP M.	**3.239 ± 0.055** -----	**2.347 ± 0.022** -----	**3.464 ± 0.323** -----	**2.739 ± 0.024** -----
C24:0	HP M.	**4.821 ± 0.356** 3.08 ± 1.07	**3.163 ± 0.068** 3.64 ± 0.67	4.488 ± 0.055 3.55 ± 0.01	1.017 ± 0.011 4.47 ± 0.09
ΣSFA	HP M.	**47.900 ± 0.393** 9.283 ± 0.829	**49.774 ± 0.452** 9.319 ± 0.610	**44.293 ± 1.039** 8.379 ± 0.033	**50.609 ± 0.429** 10.062 ± 0.085
C14:1	HP M.	**1.524 ± 0.039** -----	**0.887 ± 0.052** -----	1.362 ± 0.091 -----	1.194 ± 0.095 -----
C15:1	HP M.	**0.887 ± 0.128** **18.617 ± 0.523**	**1.919 ± 0.099** **15.522 ± 1.409**	**0.415 ± 0.069** 18.328 ± 0.004	**0.611 ± 0.012** 16.521 ± 0.007
C16:1	HP M.	**2.614 ± 0.154** **3.598 ± 0.402**	**6.284 ± 0.101** **4.926 ± 0.009**	**4.228 ± 0.384** **4.942 ± 0.004**	**6.531 ± 0.029** **4.919 ± 0.005**
C17:1	HP M.	5.615 ± 0.352 -----	5.070 ± 0.042 -----	**7.263 ± 0.700** -----	**5.426 ± 0.131** -----
C18:1n9t	HP M.	**2.108 ± 0.073** -----	**3.621 ± 0.191** -----	**3.066 ± 0.094** -----	**4.293 ± 0.055** -----
C18:1n9c	HP M.	**6.441 ± 0.167** **27.899 ± 0.045**	**6.841 ± 0.123** **29.447 ± 0.031**	**8.984 ± 0.584** **31.522 ± 0.005**	**7.741 ± 0.014** **27.430 ± 0.008**
C20:2	HP M.	**6.030 ± 0.217** **1.531 ± 0.142**	**4.716 ± 0.080** **2.291 ± 0.406**	**6.414 ± 0.246** **1.938 ± 0.023**	**4.352 ± 0.032** **1.828 ± 0.006**
C22:1n9	HP M.	**3.240 ± 0.030** **3.073 ± 0.082**	**2.163 ± 0.109** **2.378 ± 0.123**	**2.337 ± 0.174** **2.689 ± 0.035**	**2.872 ± 0.029** **2.537 ± 0.007**
C20:1	HP M.	----- **4.748 ± 0.133**	----- **3.065 ± 0.022**	----- **2.869 ± 0.035**	----- **3.063 ± 0.020**
C22:2	HP M.	**3.721 ± 0.003** -----	**1.822 ± 0.080** -----	**3.393 ± 0.536** -----	**1.328 ± 0.005** -----
C24:1	HP M.	**2.173 ± 0.122** -----	**1.487 ± 0.198** -----	**1.789 ± 0.120** -----	**1.066 ± 0.014** -----
ΣMUFA	HP M.	34.809 ± 0.48 59.465 ± 0.211	34.809 ± 0.480 57.629 ± 1.492	34.809 ± 0.270 **62.288 ± 0.042**	34.809 ± 0.480 **56.298 ± 0.020**
C20:3n3	HP M.	----- **1.648 ± 0.120**	----- **4.093 ± 0.074**	----- **1.892 ± 0.047**	----- **4.115 ± 0.049**
C22:6n3	HP M.	**2.511 ± 0.110** 1.800 ± 0.082	**1.246 ± 0.100** 1.821 ± 0.068	1.731 ± 0.929 **1.954 ± 0.060**	1.244 ± 0.324 **1.821 ± 0.068**
C18:2n6t	HP M.	**3.509 ± 0.36** **5.527 ± 0.476**	**1.838 ± 0.074** **4.522 ± 0.453**	**2.976 ± 0.353** 4.610 ± 0.017	**1.457 ± 0.040** 3.855 ± 0.025
C18:2n6c	HP M.	**3.551 ± 0.021** 22.241 ± 0.431	**3.824 ± 0.077** 22.617 ± 0.535	**3.562 ± 0.015** **20.877 ± 0.058**	**2.604 ± 0.169** **23.850 ± 0.021**
C18:3n6	HP M.	5.327 ± 0.436 -----	5.148 ± 0.106 -----	5.696 ± 0.303 -----	5.277 ± 0.004 -----
C20:3n6	HP M.	**2.850 ± 0.132** -----	**3.708 ± 0.015** -----	**2.491 ± 0.067** -----	**3.400 ± 0.104** -----
ΣPUFA	HP M.	**17.748 ± 0.361** 31.252 ± 0.721	**15.763 ± 0.211** 33.052 ± 0.987	**16.456 ± 0.541** **29.333 ± 0.075**	**13.977 ± 0.286** **33.640 ± 0.067**
Σn-3PUFA	HP M.	**2.511 ± 0.110** **3.484 ± 0.202**	**1.246 ± 0.200** **5.913 ± 0.008**	1.731 ± 0.929 **3.846 ± 0.101**	1.244 ± 0.324 **5.935 ± 0.023**
Σn-6PUFA	HP M.	**15.236 ± 0.262** 27.768 ± 0.763	**14.517 ± 0.214** 27.138 ± 0.980	**14.725 ± 0.392** **25.487 ± 0.042**	**12.733 ± 0.094** **27.705 ± 0.046**
n3/n6	HP M.	**0.165 ± 0.007** **0.126 ± 0.009**	**0.086 ± 0.008** **0.218 ± 0.008**	0.119 ± 0.065 **0.151 ± 0.004**	0.098 ± 0.026 **0.214 ± 0.001**
ΣHUFA	HP M.	8.972 ± 0.183 22.241 ± 0.431	8.972 ± 0.183 22.617 ± 0.535	9.258 ± 0.311 **20.877 ± 0.058**	8.972 ± 0.183 **23.850 ± 0.021**

HP: hepatopancreas; M: muscle. Bold black font indicates a significant difference.
